# Therapeutic targeting of voltage-gated sodium channel Na_V_1.7 for cancer metastasis

**DOI:** 10.3389/fphar.2024.1416705

**Published:** 2024-07-09

**Authors:** Piyasuda Pukkanasut, Renata Jaskula-Sztul, Juan Carlos Gomora, Sadanandan E. Velu

**Affiliations:** ^1^ Department of Chemistry, The University of Alabama at Birmingham, Birmingham, AL, United States; ^2^ Department of Surgery, The University of Alabama at Birmingham, Birmingham, AL, United States; ^3^ O’Neal Comprehensive Cancer Center, The University of Alabama at Birmingham, Birmingham, AL, United States; ^4^ Departamento de Neuropatología Molecular, Instituto de Fisiología Celular, Universidad Nacional Autónoma de México, Mexico City, Mexico

**Keywords:** cancer, metastasis, voltage-gated sodium channel, Nav1.7, therapeutic targeting, cell invasion, cell migration, cell viability

## Abstract

This review focuses on the expression and function of voltage-gated sodium channel subtype Na_V_1.7 in various cancers and explores its impact on the metastasis driving cell functions such as proliferation, migration, and invasiveness. An overview of its structural characteristics, drug binding sites, inhibitors and their likely mechanisms of action are presented. Despite the lack of clarity on the precise mechanism by which Na_V_1.7 contributes to cancer progression and metastasis; many studies have suggested a connection between Na_V_1.7 and proteins involved in multiple signaling pathways such as PKA and EGF/EGFR-ERK1/2. Moreover, the functional activity of Na_V_1.7 appears to elevate the expression levels of MACC1 and NHE-1, which are controlled by p38 MAPK activity, HGF/c-MET signaling and c-Jun activity. This cascade potentially enhances the secretion of extracellular matrix proteases, such as MMPs which play critical roles in cell migration and invasion activities. Furthermore, the Na_V_1.7 activity may indirectly upregulate Rho GTPases Rac activity, which is critical for cytoskeleton reorganization, cell adhesion, and actin polymerization. The relationship between Na_V_1.7 and cancer progression has prompted researchers to investigate the therapeutic potential of targeting Na_V_1.7 using inhibitors. The positive outcome of such studies resulted in the discovery of several inhibitors with the ability to reduce cancer cell migration, invasion, and tumor growth underscoring the significance of Na_V_1.7 as a promising pharmacological target for attenuating cancer cell proliferation and metastasis. The research findings summarized in this review suggest that the regulation of Na_V_1.7 expression and function by small molecules and/or by genetic engineering is a viable approach to discover novel therapeutics for the prevention and treatment of metastasis of cancers with elevated Na_V_1.7 expression.

## 1 Introduction

According to 2023 statistics provided by American Cancer Society, cancer is the second leading cause of death in the US. The survival rate of cancer patients depends on the stage of cancer at the time of detection. In fact, once metastasis occurs, the patient survival rate drops significantly (Guan, 2015; American Cancer Society, 2023; Siegel et al., 2023). Cancer metastasis is defined as the translocation of primary tumor cells to distant organs through the circulatory system to form secondary tumors. This process also known as the invasion metastasis cascade, is a critical stage in cancer progression (Chaffer and Weinberg, 2011; Lambert et al., 2017; Zhuyan et al., 2020). Invasion metastasis cascade can be broken down into five main steps: i) dissemination of cancer cells associated with epithelial-mesenchymal transition (EMT) and extracellular matrix (ECM) degradation, ii) intravasation of cancer cells into the circulatory system, iii) survival of circulating cancer cells in the bloodstream, iv) extravasation of cancer cells to penetrate the blood vessel wall, and v) colonization of cancer cells at distant organs (Lambert et al., 2017; Zhuyan et al., 2020). Factors that facilitate cancer metastasis include specific gene mutations (Fares et al., 2020), EMT (Mittal, 2018), ECM environment (Elgundi et al., 2019), immune system (Gonzalez et al., 2018), tumor microbiome (Fares et al., 2020), and soluble signaling molecules such as growth factors and cytokines (Fares et al., 2020). However, our understanding about the mechanisms that drive the metastatic process is still limited.

Currently, metastatic cancer is treated by controlling tumor growth by surgery followed by radiotherapy, targeted therapy, or chemotherapy. However, these therapeutic options are not very effective as none of these directly constrain the cancer from spreading to different organs (Esposito et al., 2021). Even though, the recent immunotherapy seems to be a promising approach to treat metastasis (Tan et al., 2020; Edwards et al., 2021), treatment cost is considerably high and the outcomes vary among cancer types (Esfahani et al., 2020; Gupta and Shukla, 2022) with an average response rate of only 20%–40% (Sharma et al., 2017). Therefore, metastasis remains a major cause of cancer deaths (Seyfried and Huysentruyt, 2013). To develop effective treatments that can aim to halt or slowdown cancer metastasis, the intricate molecular pathways involved in the metastatic process need to be investigated and novel drug targets need to be identified. One such novel drug target is voltage-gated sodium channels (VGSCs).

The role of VGSCs in cancer cell migration and invasiveness has been the target of many recent investigations (Roger et al., 2006; Gillet et al., 2009; House et al., 2010; Brisson et al., 2011; Brackenbury, 2012; Brisson et al., 2013; Rao et al., 2015; Mao et al., 2019; Lopez-Charcas et al., 2021; Li et al., 2022). VGSCs were first discovered in 1952 by Hodgkin and Huxley (Hodgkin and Huxley, 1952) and the existence of nine of its subtypes, Na_V_1.1-Na_V_1.9 have been confirmed so far. VGSCs were first known for their ability to initiate and propagate action potentials in nerve conduction and muscle contraction (Abdul Kadir et al., 2018). Evidence from the study of glia cell indicates that VGSCs could help maintain the ionic homeostasis in conjugation with Na^+^/K^+^ -ATPase (Sontheimer et al., 1994). The inhibition of VGSCs by TTX reduced intracellular sodium concentration [Na^+^], consequently leading to a significant decrease in Na^+^/K^+^ -ATPase activity (Sontheimer et al., 1994). More recently, certain VGSC subtypes were found to be overexpressed in various cancers (Djamgoz et al., 2019; Lopez-Charcas et al., 2021). For example, Na_V_1.5 was found in breast (Gillet et al., 2009; Brisson et al., 2011; Brisson et al., 2013; Driffort et al., 2014; Dutta et al., 2018) ovarian, colon (Lopez-Charcas et al., 2023), leukemia (Fraser et al., 2004; Carrithers et al., 2007; Huang et al., 2015), and brain (Xing et al., 2014) cancers; Na_V_1.6 was found in cervical (Lopez-Charcas et al., 2018), colorectal (Lin et al., 2019), follicular thyroid (Li et al., 2022) melanoma (Carrithers et al., 2009), and leukemia (Carrithers et al., 2009); and Na_V_1.7 in prostate (Diss et al., 2005; Brackenbury, 2012), gastric (Xia et al., 2016), endometrial (Liu et al., 2019), colorectal (Pan et al., 2017), lung (Campbell et al., 2013), and medullary thyroid cancer (Pukkanasut et al., 2023). In addition, the activity of these VGSC subtypes was found to be significantly higher during the development of cancer (Tokuoka and Morioka, 1957; Binggeli and Weinstein, 1986).

This review focuses on the expression and function of Na_V_1.7 and its possible role in the progression of various cancer types. We have also summarized the studies on Na_V_1.7 binding sites, the use of Na_V_1.7 inhibitors in cancers and provided an overview of the possible mechanisms of action for Na_V_1.7 mediated cancer cell proliferation, migration and invasion.

## 2 Na_V_1.7 overexpression in neuronal and cancer cells

Among the nine VGSC subtypes, Na_V_1.7 (*SCN9A* encoded gene) has been identified as a remarkable drug target for pain therapy (Dormer et al., 2023). Na_V_1.7 is predominantly expressed in peripheral nervous system (PNS) especially in all types of dorsal root ganglion (DRG) neurons (William et al., 2005; Hameed, 2019) which have an important role in operating nociceptive signaling for pain and has been reported to be associated with inherited pain disorders (Dib-Hajj et al., 2010; Ahuja et al., 2015; Dormer et al., 2023). The mutations that upregulate the function of Na_V_1.7 cause severe neuropathic pain such as inherited erythromelalgia, and paroxysmal extreme pain disorder (Dormer et al., 2023), whereas mutations that cause loss of Na_V_1.7 function result in congenital indifference to pain (CIP) (Goldberg et al., 2007; Dib-Hajj et al., 2013).

More recently, VGSCs have been found to be expressed in several human carcinomas (Angus and Ruben, 2019) and the evidence suggests that the predominant subtypes expressed in human carcinoma tissues are Na_V_1.5 and Na_V_1.7 (Djamgoz et al., 2019; Horne et al., 2021). To date, several different types of cancers have been reported to have high expression of Na_V_1.7. This includes human prostate biopsies (Diss et al., 2005), human prostate cancer cell lines, PC3, and LNCaP (Diss et al., 2001; Shan et al., 2014; Luiz and Wood, 2016); human lung cancer cell lines, Calu-1, H23 and H-460 (Roger et al., 2007; Campbell et al., 2013); and human ovarian cancer cell lines, Caov-3, SKOV-3 and Anglne (Gao et al., 2010). In addition, Na_V_1.7 is found to be overexpressed in gastric cancer tissues and cell lines, BGC-823 and MKN-28 (Xia et al., 2016), in endometrial cancer cells and biopsies (Liu et al., 2019), in primary malignant pleural mesothelioma (MPM) cells obtained from patient specimens (Fulgenzi et al., 2006), in human neuroblastoma cell line, SH-SY5Y (Vetter et al., 2012), and in human medullary thyroid cancer tissues and cell lines, MZ-CRC-1 (highly metastatic) and TT (primary tumor) (Pukkanasut et al., 2023). Additionally, lower expression of Na_V_1.7 is detected in breast cancer tissues and cell line, MDA-MB-231 (Fraser et al., 2005), cervical cancer biopsies and primary cultures (Hernandez-Plata et al., 2012), and lymphocyte leukemia cell lines, Jurkat, MOLT-4, and Ball (Fraser et al., 2004; Huang et al., 2015).

## 3 Nav1.7 structure

**FIGURE 1:**
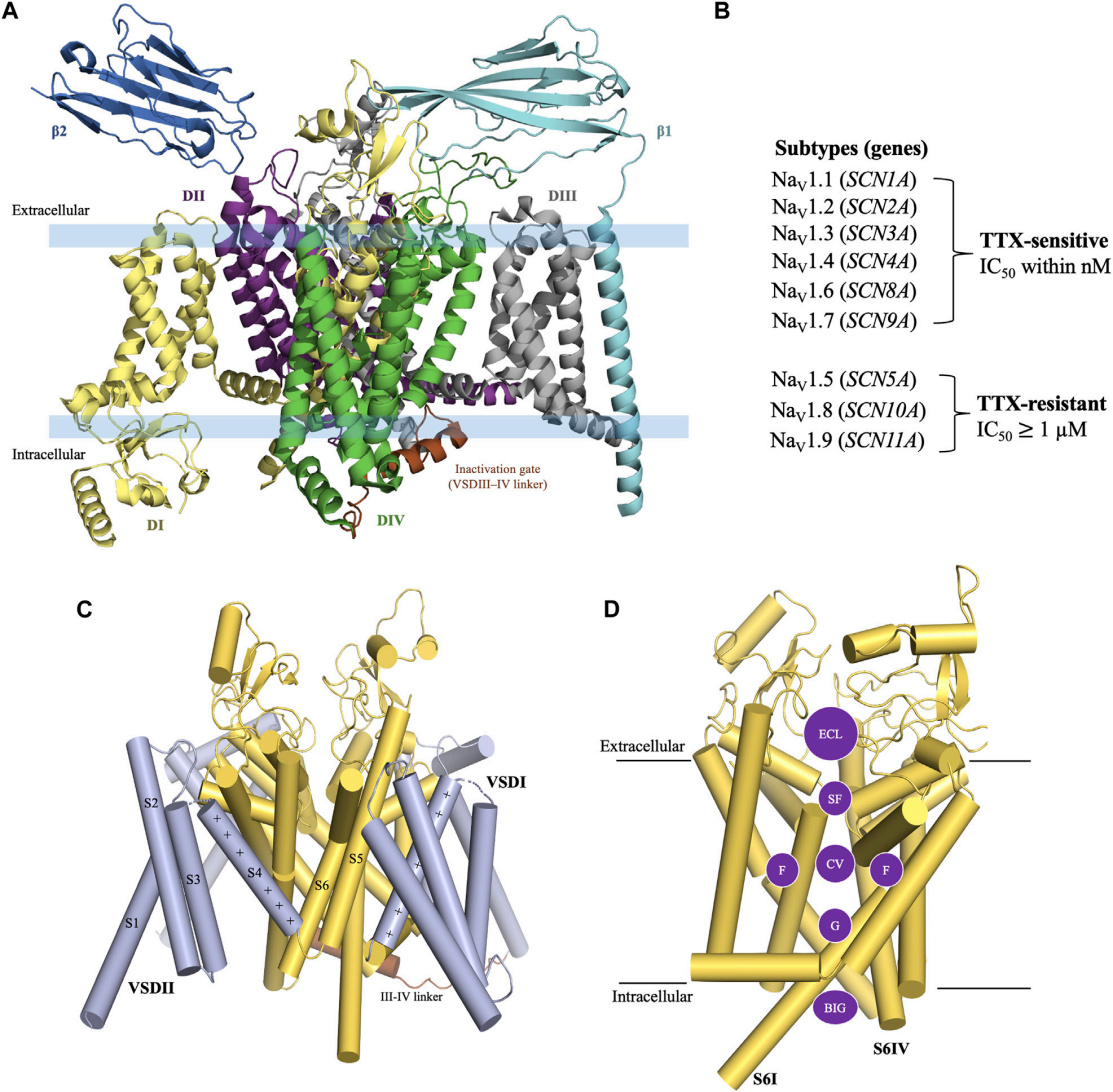
General structure of voltage-gated sodium channel. **(A)** The structure of VGSC consists of an α subunit with four homologous Domain (DI-DIV) and one or two β auxiliary subunits. **(B)** TTX-sensitive and TTX-resistant voltage-gated sodium channels. **(C)** Construction of the α subunit. Each Domain of the α-subunit consists of six transmembrane segments. S1-S4 are a part of the voltage sensing domain and S5-S6 are a part of the pore forming domain (PD). **(D)** Pore forming domain is divided into five regions i) an extracellular loop (ECL); ii) a selectivity filter (SF); iii) a central cavity (CV) which contains fenestration sites (F); iv) an intracellular activation gate (G); and v) the region beneath the intracellular gate (BIG). Structure figures were prepared in PyMol 2.4.2 from the PDB 8thh (Wu et al., 2023)

The structure of eukaryotic VGSCs is conserved with >50% sequence homology across the nine subtypes (de Lera Ruiz and Kraus, 2015; Sanchez-Sandoval et al., 2023). The structure of VGSC consists of an alpha (α) subunit which contains four large homologous Domains (DI-DIV) [Figure 1A] with approximately 2000 amino acid residues (MW ∼260 kDa) (William et al., 2005), and one or two beta (β) auxiliary subunits which consist of approximately 220 amino acid residues per unit (MW 30–40 kDa) (de Lera Ruiz and Kraus, 2015; Deuis et al., 2017; Salvage et al., 2020; Sayers et al., 2022). Each domain of the α-subunit contains six transmembrane segments S1-S6, in which segments S1-S4 are voltage-sensing domains (VSD) and the segments S5 and S6 contribute to the pore forming domain (PD) (Sula et al., 2017) [Figure 1C]. The PD consists of five regions i) an extracellular loop (ECL), ii) a selectivity filter (SF) also known as the pore region which is located at the extracellular end of the channel, iii) a central cavity (CV) which contains a fenestration site, a receptor site for local anesthetics (LA) (Shen et al., 2017; Wu et al., 2023), iv) an intracellular activation gate (G), and v) the region beneath the intracellular gate (BIG) [Figure 1D] (Shen et al., 2017; Buyan et al., 2018; Wu et al., 2023).

**FIGURE 2:**
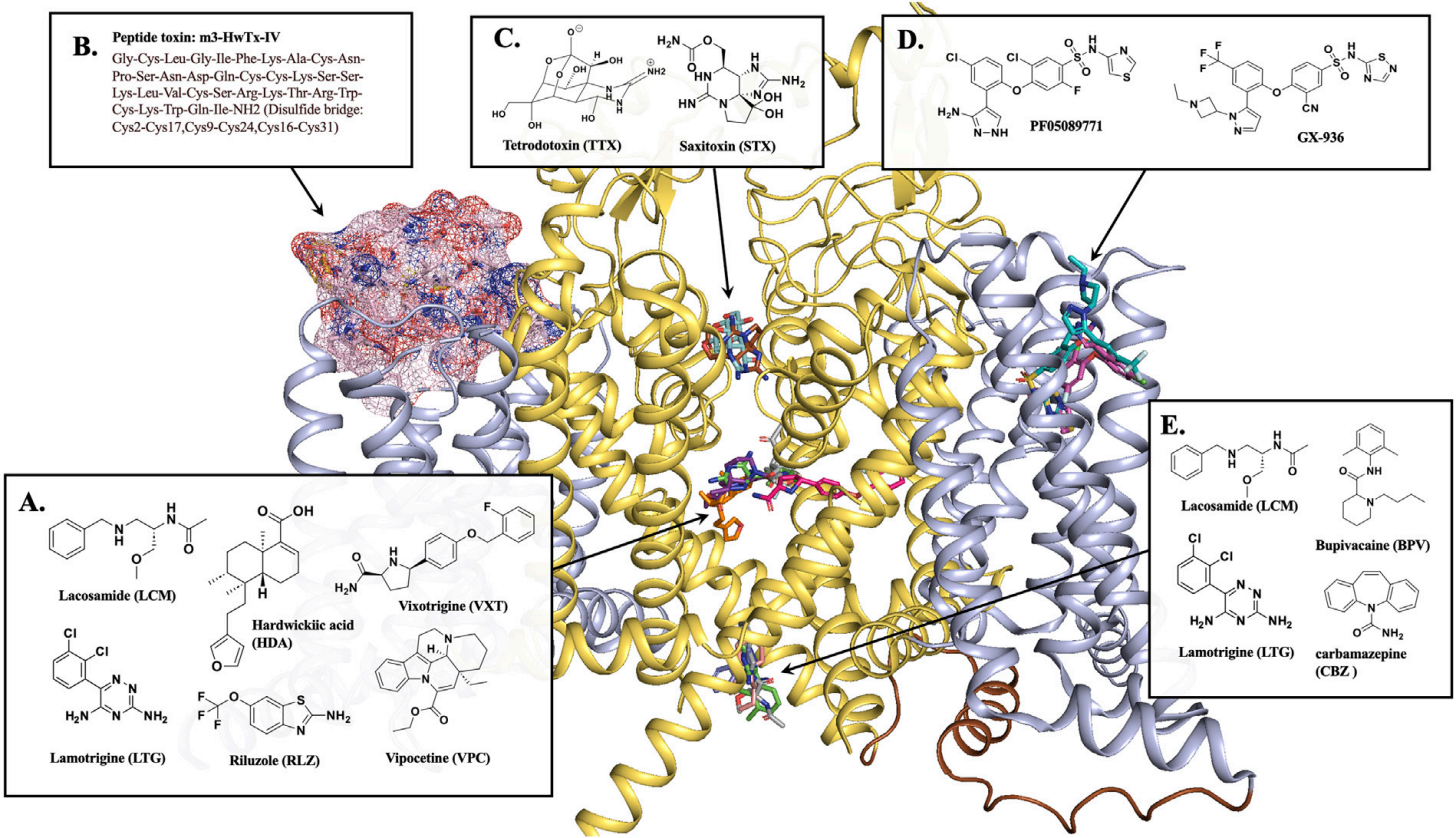
Na_V_1.7 binding sites for different inhibitors. **(A)** Most inhibitors occupy the CV region of the central pore of the channel. The CV site inhibitors, HDA (orange stick, PDB 8j4f), VXT (hot pink stick, PDB 8i5y), RLZ (beige stick, PDB 8thg), and VPC (purple stick, PDB 8i5x). **(B)** Peptide toxin, m3-HwTx-IV (pink surface) bound at VSDII, PDB 7k48. **(C)** Sodium channel blockers, TTX (pale cyan stick, PDB 6j8j) and STX (brown stick, PDB 6j8h) block the selectivity filter. **(D)** Na_V_1.7 selective inhibitors PF05089771 (magenta stick, PDB 8i5g) and GX-936 (cyan stick, PDB 5ek0) bind at VSDIV. **(E)** BPV (light pink stick, PDB 8i5b) and CBZ (blue stick, PDB 8s9c) are located at the BIG site. LCM (gray sticks, PDB 8s9b) and LTG (green sticks, PDB 8thh) is a dual-inhibitor binding to both CV region and BIG site. This figure was prepared in PyMol 2.4.2.

The VGSC subtypes were originally classified in terms of their sensitivity to TTX, a cyclic guanidinium molecule obtained from puffer fish (Noguchi et al., 2006). The subtypes Na_v_1.1, Na_v_1.2, Na_v_1.3, Na_v_1.4, Na_v_1.6, and Na_v_1.7 are known as TTX-sensitive as they require only a low nanomolar concentration of TTX for their blockade. Whereas, the subtypes Na_v_1.5, Na_v_1.8, and Na_v_1.9 are known as TTX-resistant as they require micromolar concentrations of TTX for their blockade [Figure 1B] (de Lera Ruiz and Kraus, 2015). TTX blocks the channels by binding to the outer vestibule near selectivity filter (SF) site of the channel [Figure 2C] (Elliott and Elliott, 1993; Huang et al., 2012; Chen and Chung, 2014; Ahuja et al., 2015). The blockade by TTX is not state-dependent or use-dependent in contrast to LAs, which bind to the inner pore region of the channel and have higher binding affinity when the channel is being used during the opening and inactivated states compared to the resting state of channel (Ragsdale et al., 1994; Wang and Strichartz, 2012). Therefore, LAs are state-dependent or use-dependent blockers of VGSCs.

## 4 Na_V_1.7 drug binding sites

The X-ray crystal structure of human Na_V_1.7 co-crystallized with a selective antagonist, aryl sulfonamide, GX-936 was first reported in 2015 (PDB 5EK0) (Ahuja et al., 2015). This structure revealed the interactions of GX-936 with the fourth gating charge (R4) of VSDIV at the extracellular region of Na_V_1.7 (Figure 2D) (Ahuja et al., 2015). The investigation of the inhibitory activity of aryl sulfonamide class antagonists utilized GX-674, an analog of GX-936. The potency of GX-674 against Na_V_1.7 was about 100,000 times higher than Na_V_1.5. The mutant R1608A showed a 2700-fold decrease in the activity of Na_V_1.7 (Ahuja et al., 2015), suggesting that R1608 residue is critical for the binding GX-674 to Na_V_1.7. Indeed, there might be sufficient differences in amino acid residues for drug selectivity around voltage sensor region (S1-S4) of VSDIV domain among VGSCs. The selectivity for VSDIV binding was previously observed in the modification of Na_V_1.3’s VSDIV by generating chimeras and mutants replacing S1–S4 of Na_V_1.3 with the same segment of Na_V_1.5. This led to a 500-fold decrease in the potency of the selective Na_V_1.3 inhibitor, ICA-121431. Based on this study, the key residues in Na_V_1.3 VSDIV were determined to be S1510, R1511 and E1559 which correspond to residues, Y1537, W1538 and D1586 in Na_V_1.7 (McCormack et al., 2013).

Further studies on VSDIV were conducted using a selective Na_V_1.7 inhibitor, PF-04856264 (McCormack et al., 2013; Swain et al., 2017). Upon switching the three residues, Y1537, W1538 and D1586 in Na_V_1.7, with the matching Na_V_1.3 residues, S1510, R1511 and E1559 on the VSDIV resulted in a significant loss of potency. However, these mutations did not affect the sensitivity to TTX or the local anesthetic tetracaine (McCormack et al., 2013), suggesting that the residues Y1537, W1538 and D1586 are critical for the selectivity of PF-04856264 to Na_V_1.7 and sodium channel blockers, such as TTX and local anesthetics bind to different sites within the channel (Ragsdale et al., 1994; Chen and Chung, 2014). The modification of PF-04856264 led to the discovery of a more potent and safer clinical candidate of NaV1.7 inhibitor, PF-05089771 [Figure 2D] (Swain et al., 2017).

In 2019, Cryo-EM structures of human Na_V_1.7 complexed with a peptide gating modifier, Pro-toxin II (spider toxin) and TTX (PDB 6j8j) and the same channel with the peptide gating modifier, huwentoxin-IV (spider toxin) and saxitoxin (STX) were published (PDB 6j8h) for the first time (Shen et al., 2019). These structures clearly proved that the binding site of TTX and STX within Na_V_1.7 is located at the extracellular vestibule near the selectivity filter [Figure 2C]. However, the binding site for Pro-toxin II was found to be above VSDII and VSDIV and the binding site for huwentoxin-IV was found to be in VSDII segment as suggested by the blobs of electron densities in the previous structure studies with a 3.2 Å resolution (Shen et al., 2019).

Later in 2021, Wisedchaisri *et al* were able to obtain the Cryo-EM structure of the mutated form of Na_V_1.7 which trapped the resting state of the channel complexed with a huwentoxin-IV analog, m3-HwTx-IV (PDB 7k48) [Figure 2B] (Wisedchaisri et al., 2021). This structure further supported the huwentoxin-IV binding site to be in VSDII region. The toxin analog, m3-HwTx-IV showed high affinity for Na_V_1.7 resting state. The key interactions are at the extracellular loop region of S3-S4 between the negatively charged residues, E811, D816 and E818 of Na_V_1.7 and the positively charged K27 and K32 residues of m3-HwTx-IV. In addition, several hydrophobic residues in S3-S4, ^812^LFLAD^816^ displayed hydrophobic and van der Waals interactions with various residues of m3-HwTx-IV such as I5, F6, T28, W30, K32 and Q34. It is to be noted that the residues F813 could be responsible for Na_V_1.7 selectivity as this residue is unique to Na_V_1.7. In addition to the interactions from S3-S4, the residues E753 and N763 from S1-S2 also made a close interactions with K27 and R29 residues of m3-HwTx-IV (Wisedchaisri et al., 2021).

More recently, the Cryo-EM structures of human Na_V_1.7 complexed with multiple small-molecule inhibitors were reported (Wu et al., 2023). The inhibitors presented in this publication are a local anesthetic bupivacaine (BPV); anticonvulsants lacosamide (LCM) and carbamazepine (CBZ); and antinociceptive compounds hardwickiic acid (HDA), vinpocetine (VPC), and vixotrigine (VXT) (Wu et al., 2023). The structures showed that all six inhibitors bind to the intracellular end of S6 segment which is highly conserved among nine VGSC subtypes (Kitano and Shinozuka, 2022; Wu et al., 2023). BVP, LCM and CBZ occupied the same BIG site [Figure 2E] where L964, I1457, and I1756 are involved in the binding for all three drugs. Notably, LCM was found to bind at a different location at the upper CV region closer to S6DI and S6DIV. The benzyl ring of the inhibitor participated in hydrophobic interactions with the residues S1697, I1744, F1748, V1751, and V1752 while the two amide groups of the inhibitor were engaged in two H-bonding interactions with T1696 and Q360 [Figure 2A]. The inhibitors HDA, VPC and VXT bind to the fenestration site of the Na_V_1.7 channel [Figure 2A]. The inhibitors HDA and VPC are bound between S6DIII and S6DIV interacting with T1404, W1332, T1448, L1449, F1452, S1697, I1744, and F1748. The inhibitor VXT penetrated the channel through the cleft formed by S6DI and S6DIV to bind in the fenestration site where it interacted with residues F1692, T1695, T1696, I386, F387, F391, V1751, and Y1755. Interestingly, the presence of the inhibitors BVP, LCM and CBZ at the BIG site prevented access to fenestration sites for inhibitors HDA, VPC and VXT. This might be due to a conformational shift from α to π position of S6IV caused by the drug-binding at BIG (Huang et al., 2022) resulting in the closure of the fenestration site (Wu et al., 2023).

Also in 2023, the cryo-EM structures of human Na_V_1.7 bound to two FDA approved drugs, riluzole (RLZ) and lamotrigine (LTG) were reported (Huang et al., 2023). Both drugs occupied the CV site near S6DIII and S6DIV [Figure 2A]. Unlike RLZ, which was binding only in the CV region, LTG was also found to bind in the BIG site [Figure 2E], displaying a dual-pocket inhibition mechanism similar to LCM. At CV region, the main interactions of RLZ were hydrophobic interactions of the benzothiazole ring of the drug with residues Q360, F391, T1404, T1695, T1696, S1697, I1744 and F1748. LTG had similar hydrophobic interactions through its phenyl ring with the residues F391, S1697, F1748, V1751, and V1752. LTG also displayed H-bonding interactions of its triazine ring with the amide carbonyl backbone of the residues, A1403, K1460 and S1445. Even though both drugs had different binding modes, F1748 was found to be an important conserved residue for the LA binding.

The structure-based design of the subtype selective VGSC inhibitors has been challenging in the past due to the lack of accurate structure information of the specific channels and their binding sites. However, the recent advances in Cryo-EM technology in solving the structures of eukaryotic VGSC subtypes has made a huge impact on the design and discovery of inhibitors that are specific to channel subtypes, thereby increasing the potential success rate of VGSC targeted drug discovery (Mollica et al., 2016; Boezio et al., 2018; Jiang et al., 2022; Mahapatra et al., 2022; Sadybekov and Katritch, 2023).

## 5 Na_V_1.7 expression in different cancer types and implication of its regulation by small molecules, siRNA and shRNA

**FIGURE 3:**
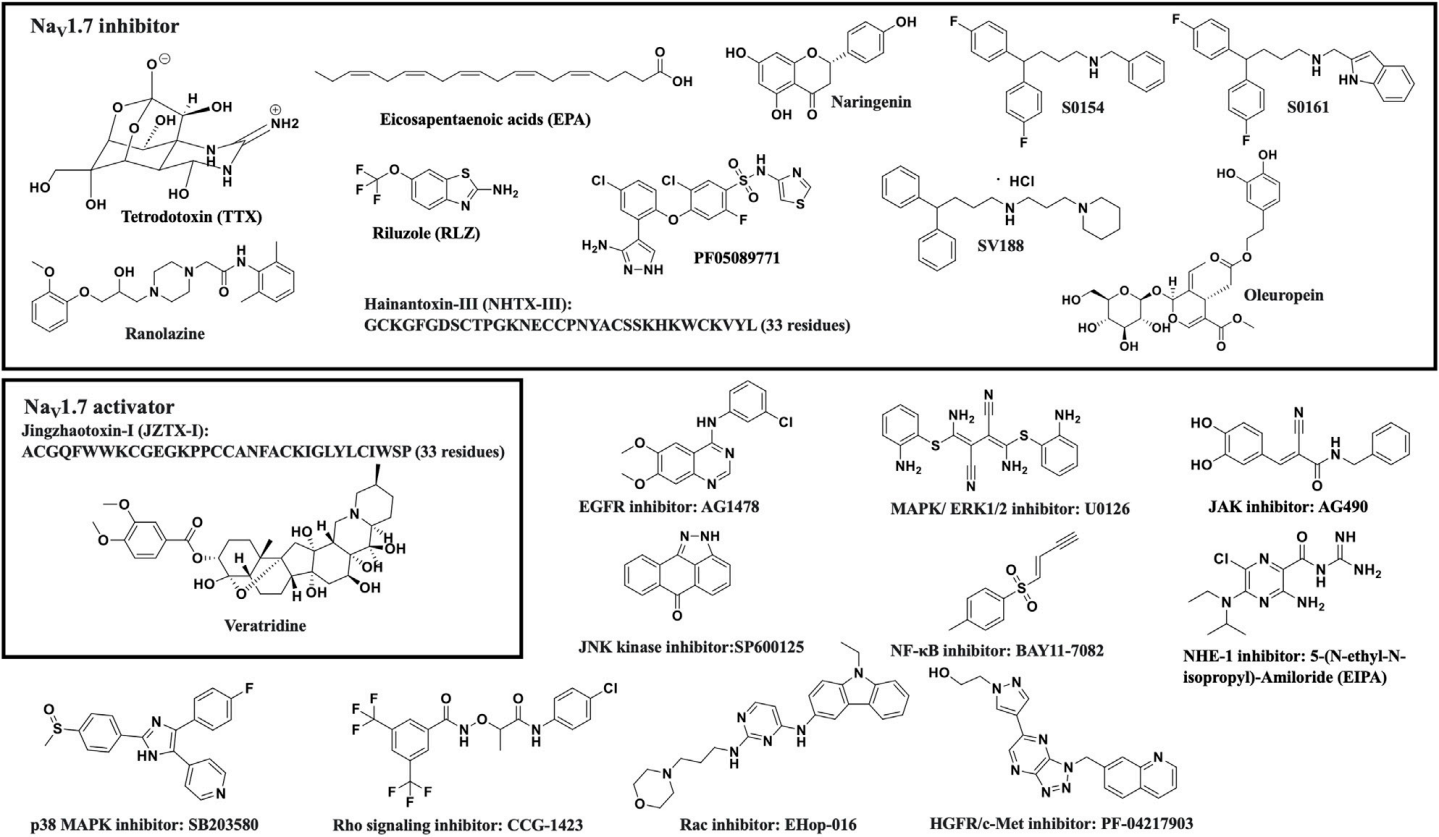
Structures of compounds used in the studies of Na_V_1.7 in cancers.

Several recent studies have reported the overexpression of Na_V_1.7 and the effects of its inhibition or silencing in different cancer types. The results of these studies suggest that Na_V_1.7 expression and function are involved in driving the metastatic cell functions such as cell migration, invasion, viability, and apoptosis. A brief overview of the association of Na_V_1.7 to these activities is provided in Table 1. Table 1 and the chemical structures of the compounds used in the studies of Na_V_1.7 in cancer are shown in Figure 3.

**TABLE 1:** Summary of Na_V_1.7 expression in different cancer types, Na_V_1.7 inhibitors, their effects on sodium currents, gene expression and anti-cancer activities.

Cancer types	Subjects	Compounds/drugs and doses	Effects on INa	Effect on genes and/or proteins	Anti-cancer effects
Primary malignant pleural mesothelioma (MPM)	MPM cells derived from patients’ specimens	TTX, 2 μM	IC_50_ = 16 nM	N/A	∼15% reduction in cell motility (Fulgenzi et al., 2006)
Prostate cancer (PCa)	MAT-LyLu	TTX, 1 μM	56% reduction in *I*_Na_ density	Degraded Na_V_1.7 mRNA expression	61% reduction in migration (Brackenbury and Djamgoz, 2006)
	Rat models	TTX, 200 nM	N/A		44% reduction in the number of metastases (Yildirim et al., 2012)
	MAT-LyLu and PC-3	TTX, 10 μM Eicosapentaenoic acid (EPA), 30 μM	TTX: I_Na_ IC_50_ = 7 nM EPA: I_Na_ IC_50_ = 6 μM	Degraded Na_V_1.7 mRNA expression	TTX: 60% reduction in cell invasion EPA: 50% reduction in cell invasion Significantly reduced endocytosis activity (Nakajima et al., 2009)
	PC-3 sh-*SCN9A and* sh-*SCN8A*	N/A	N/A	N/A	PC-3 sh-*SCN9A and* sh-*SCN8A:* 60% reduction in cell invasion PC-3 sh-*SCN9A:* 40% reduction in cell invasion Significantly reduced endocytosis activity (Nakajima et al., 2009)
	MAT-LyLu	Naringenin, 5 and 10 μM and TTX, 600 nM	N/A	Degraded Na_V_1.7 mRNA expression	∼50% reduction in cell invasion for all treated groups (Gumushan and Akgun, 2018)
	PC-3, LnCaP, and DU145 Mice xenograft	S0154, 10 μM (*in-vitro*), 5 mg/kg (*in vivo*) S0161, 10 μM (*in-vitro*), 5 mg/kg (*in vivo*)	The treatments for 30 min increased intracellular Na^+^ concentration by ∼70%	Degraded Na_V_1.7 Na_V_1.6, MMP-2 and MMP-9 basal expression	Up to 30% reduction in glucose uptake up to 95% inhibition in cell invasion S0154 at 20 μM increased apoptosis Slow down tumor growth rates S0161: reduced tumor weight (Wang et al., 2019)
	Rat models PCa tissues	Ranolazine, 2.5 and 5 μM	N/A	N/A	Reduction in lung metastases Reduced Na_V_1.7 expression in lung metastases Na_V_1.7 expression levels are associated with tumor progression (Bugan et al., 2019)
	MAT-LyLu	JZTX-I (Na_V_1.7 activator), 5 μM HNTX-III (Na_V_1.7 inhibitor), 5 μM	JZTX-I: increased *I*_Na_ by 20% HNTX-III: reduced *I*_Na_ by 80%	JZTX-I increased RhoA and Rac1 basal expression while HNTX-III degraded both basal expression	JZTX-I: induced cell migration and invasion by 26% and 28% HNTX-III: inhibited cell migration and invasion by 39% and 41%
	MAT-LyLu sh-*SCN9A*	N/A	N/A		sh-*SCN9A* 60% reduction in cell migration and invasion (Chen et al., 2019)
	MAT-LyLu	Oleuropein, 1, 5 and 10 μM	N/A	Degraded Na_V_1.7 mRNA expression	Up to 18% reduction in cell migration (Aktas and Ayan, 2021)
	MAT-LyLu	TTX, 1 μM Riluzole, 5 μM Ranolazine, 20 μM	N/A	Riluzole: degraded Na_V_1.7 mRNA expression under hypoxic condition	TTX: 50% and 53% decreased in cell invasion under normoxic and hypoxic conditions Riluzole: 45% decreased in cell invasion under normoxic conditions Ranolazine: 55% and 66% decreased in cell invasion under normoxic and hypoxic conditions (Rizaner et al., 2020)
Non-small-cell lung cancer (NSCLC)	Invasive cancerous cell lines: H23, H460, Calu-1 Weakly metastatic cell line: A549 Non-cancerous cell lines: NL-20 and BEAS-2B	TTX, 1 μM (H23, H460) and 30 μM (Calu-1)	*I*_Na_ was only observed in H23, H460 and Calu-1 *I*_Na_ in H23, H460 and Calu-1 was fully blocked using TTX	N/A	Up to 50% reduction in cell invasion (Roger et al., 2007)
	H460, A549	TTX, 0.5 μM veratridine, 50 μM (Na_V_s activator)	TTX: shifted *E*_m_ to be more hyperpolarized in H460 TTX: decreased the internal sodium concentration by 50%	N/A	TTX: 40% reduction in cell invasion Veratridine: 20% increase in cell invasion
	H460 sh-SCN9A NSCLC tissues		H460 sh-*SCN9A*: completely demolished *I*_*N*a_ in H460	5-fold decreased in Na_V_1.7 mRNA expression	sh-*SCN9A*: 50% reduction in cell invasion Na_V_1.7 expression levels are associated with tumor progression (Campbell et al., 2013)
Ovarian cancer (OC)	Invasive cancerous cell lines: Caov-3, SKOV-3 Weakly metastatic cell line: Anglne	TTX, 30 μM	N/A	N/A	Caov-3: 62% reduction in cell migration, 57% reduction in cell invasion SKOV-3: 50% reduction in cell migration, 59% reduction in cell invasion (Gao et al., 2010)
Gastric cancer (GC)	319 GC patient samples	N/A	N/A	N/A	Strong correlation between Na_V_1.7 and NHE-1 expression
	BGC-823, MKN-28 BGC-823 sh-*SCN9A*, MKN-28 sh-*SCN9A*	TTX, 1 μM NHE-1 inhibitor 5-(N-ethyl-N-isopropyl) amiloride (EIPA), 10 μM N/A	N/A N/A	TTX and sh-*SCN9A*: Degraded NHE-1 and MACC1 mRNA expression and protein expression	TTX and sh-*SCN9A*: A reduction in cell proliferation TTX and EIPA: >60% reduction in cell invasion Increased in an extracellular pH Decreased in an intracellular pH
	Mice models sh-*SCN9A*	N/A	N/A	N/A	A reduction in tumor growth rates including the tumor weights and sizes (Xia et al., 2016)
Endometrial cancer (EC)	EC tissues	N/A	N/A	N/A	Na_V_1.7 expression levels are associated with tumor size, and tumor progression
	EC cells	TTX, 10 μM PF-05089771, 100 μM Veratridine, 100 μM	N/A	N/A	TTX and PF-05089771 significantly decreased EC cells invasion Veratridine: significantly increased cell invasion (Liu et al., 2019)
Medullary thyroid cancer (MTC)	MTC tissues	N/A	N/A	N/A	Na_V_1.7 mRNA expression was higher in MTC patient tissues compared to its normal counterparts
	Metastatic cancerous cell lines: MZ-CRC-1 Primary cancerous cell line: TT	SV188, 3 μM and 6 μM	Na_V_1.7 IC_50_ = 3.6 μM Shifted the voltage- dependence of activation of Na_V_1.7 to be more negative Displayed a use-dependent inhibitory which the inhibition reached to 77% when increasing the test pulses	Degraded NHE-1 and Na_V_1.7 mRNA expression	Up to 57% reduction in cell migration in MZ-CRC-1 and TT. Up to 52% reduction in cell invasion in MZ-CRC-1 Induced cell arrest at G0/G1 phase and decreased the number of cells at S and G2 phases (Pukkanasut et al., 2023)

### 5.1 Prostate cancer (PCa)

The first and the most widely studied cancer type to establish the relationship between Na_V_1.7 and disease progression is prostate cancer (PCa) using a MAT-LyLu rat cell line (Brackenbury and Djamgoz, 2006). The study revealed that the pretreatment of MAT-LyLu cells with 1 μM TTX or 500 nM of KT5720, a protein kinase A (PKA) inhibitor significantly reduced the sodium current (*I*_Na_) density by 56%. In addition, the TTX pretreatment had no effect on the voltage-dependence of the activation, steady-state inactivation, or sodium window current ( – 60 to – 20 mV) where the channels were partially activated and not fully inactivated. The co-pretreatment with TTX and KT5720 also significantly reduced mRNA levels of Na_V_1.7. However, the co-treatment did not show any improvement compared to individual treatments. The migration assay using MAT-LyLu cells showed that the TTX pretreatment for 48 h vs. the TTX treatment which applied during the assay reduced cell migration by 61% and 58%, respectively compared to the control. Later in 2012, Yildirim *et al* reported that the treatment of PCa rat models generated using MAT-LyLu cells with 200 nM TTX significantly reduced the metastasis lesions by 44% with no significant effect on the tumor size (Yildirim et al., 2012).

In 2009, Nakajima *et al* showed that eicosapentaenoic acid (EPA) inhibited VGSCs and reduced the metastatic cell functions such as cell invasion and endocytosis activity in PCa cell lines PC-3 and MAT-LyLu (Nakajima et al., 2009; Khan and Steeg, 2021). Quantitative RT-PCR showed that Na_V_1.6 and Na_V_1.7 were predominantly expressed in both PCa cell lines. In addition, treatment with 1 μM TTX completely abolished *I*_Na_ in these cells. TTX and EPA treatments in MAT-LyLu cells were dose dependent with IC_50_ values of 7 nM and 6 μM, respectively. Na_V_1.6 and Na_V_1.7 mRNA expression levels were significantly decreased by 9-fold after the treatments with EPA and TTX in PC-3 cells. The role of Na_V_1.6 and Na_V_1.7 in cell invasion was further analyzed by TTX and EPA treatments compared to PC-3 cells transfected with small interfering (siRNA) for *SCN8A* (Na_V_1.6) and *SCN9A* (Na_V_1.7). The results showed that the treatments with TTX and EPA significantly inhibited the cell invasion. PC-3 transfected cells with siRNAs for both *SCN8A* and *SCN9A* showed 20% greater migration inhibition compared to siRNA for *SCN9A* alone. These results suggest that both channels are participating in the invasion activity of PC-3 cells. Furthermore, endocytosis activity in PC-3 cells was examined by measuring horseradish peroxidase (HRP) uptake, the results of which showed that the cells treated with TTX and EPA as well as PC-3 transfected siRNA for *SCN9A* and *SCN8A* displayed significantly reduced HRP uptake (Nakajima et al., 2009).

In 2005, Diss *et al* showed higher Na_V_1.7 immunohistochemistry staining and mRNA expression in PCa patient biopsies compared to non-cancerous samples. (Diss et al., 2005). Later in 2013, the examination of Na_V_1.7 in tissues from patients at different disease progression stages of PCa showed distinct difference ranging from low to high expression in low and high grade tumors (Campbell et al., 2013). Consistent with the 2009 report, there were significant differences in Na_V_1.7 expression of PCa cancerous tissues vs. normal tissues (Bugan et al., 2019) suggesting a correlation between Na_V_1.7 expression and PCa progression.

The anticancer activities of a natural product, naringenin, were reported against the motility of MAT-LyLu cells in 2018 by Gumushan *et al* (Gumushan and Akgun, 2018). Treatment with naringenin at 50 μM inhibited the cell growth and induced apoptosis in MAT-LyLu cells. The treatment with 10 μM naringenin or 600 nM TTX significantly decreased Na_V_1.7 mRNA expression by 1.3-fold and 3.4-fold, respectively. Moreover, the treatments with 5 μM or 10 μM naringenin showed relatively similar inhibitory effects on the cell invasion as observed for 600 nM TTX.

In a study by Jiajia *et al* published in 2019, fifteen sodium channel inhibiting amine and amide compounds were evaluated for their inhibitory effects on PCa metastasis *in vitro* using cell invasion and wound healing assays and *in vivo* using mice tumor xenografts (Wang et al., 2019). Two secondary amine compounds S0154 and S0161 showed anti-cancer and anti-metastatic effects against the cell line with metastatic castration-resistant prostate cancer (mCRPC), PC-3. The treatment with 10 μM of S0154 or S0161 for 30 min significantly increased the internal [Na^+^] by blocking the basal Na^+^ effusion in PC-3 cells. The treatment with S0154 at 10 μM reduced Na_V_1.7 and Na_V_1.6 basal expression. Meanwhile, Na_V_1.7 was found to be more sensitive to S0161 showing reduced basal expression at 2.5 μM, while the effect on Na_V_1.6 degradation was noticed at 10 μM. The glucose uptake by PC-3, as determined using fluid-phase tracer FITC-glucose showed a significant depletion by 20%–30% after the treatment with S0154 or S0161. An increase in cell apoptosis was observed when treated with 20 μM of S0154 while no apoptosis was observed when treated with 20 μM of S0161 suggesting that the mechanism of S0161 cell viability inhibition at 5 μM was not a result of apoptosis. In addition, treatment with either S0154 or S0161 significantly inhibited PC-3 cell invasion in a dose-dependent manner. A slight reduction in cell migration was observed in wound healing assays, however, the change was not significant when treated with both compounds. Further studies in mice xenograft models demonstrated the anti-metastatic effects of S0154 and S0161. The slower tumor growth rates were observed after the treatment with both compounds at 5 mg/kg BW with no toxicity detected. After 21 days of treatment with S0161, the animals showed significant reduction in tumor weight while the treatment with S0154 showed no significant effects, indicating stronger anti-cancer and anti-metastatic effects for S0161.

Ranolazine, a known VGSC inhibiting drug used for angina treatment was evaluated for its anti-cancer, and anti-metastasis effects and the influence on Na_V_1.7 in primary tumor and metastasis in PCa rat models (Bugan et al., 2019). The treatment with ranolazine in rat models significantly reduced lung metastasis with no significant difference in tumor weight compared to the control suggesting that ranolazine has anti-metastatic but not anti-tumorigenic activity against PCa. Furthermore, Na_V_1.7 protein level in lung metastatic lesions of PCa rat models were assessed by immunostaining showed lower expression in immunoreactive cells compared to control.

The effects of peptide toxins, JZTX-I (Na_V_1.7 activator) and HNTX-III (Na_V_1.7 inhibitor) on the invasive ability of PCa MAT-LyLu cells were evaluated in 2019 by Chen *et al* (Chen et al., 2019). The study found that these two peptides modulated Na_V_1.7 activity and regulated the migration and invasion of MAT-LyLu cells. The treatment with 5 μM JZTX-I increased *I*_Na_ by 20% and induced cell migration and invasion while 5 μM treatment with HNTX-III reduced *I*_Na_ by 80% and inhibited cell migration and invasion. An additional study using knocked down Na_V_1.7 in MAT-LyLu cells showed a difference in migration and invasion between treated and non-treated cells (Chen et al., 2019). Na_V_1.7 knocked down MAT-LyLu cells showed substantially reduced cell migration and invasion activities, whereas the treatments with either of the peptides did not alter the migration and invasion. This data suggests that JZTX-I and HNTX-III specifically targeted Na_V_1.7 and altered the migration and invasion activities of MAT-LyLu cells.

Aktas *et al* reported a study of another natural product, oleuropein, a major component in the aqueous extract of olive leaves (AOLE) in voltage-gated ion channels which showed inhibitory effects against the L-type (Scheffler et al., 2008) and T-type (Marchetti et al., 2015) calcium channels and the Na_V_1.7 sodium channel (Aktas and Ayan, 2021). Oleuropein displayed an IC_50_ value of 47 μM against MAT-LyLu cells and significantly decreased cell migration in wound healing assay up to 10%. Additionally, the treatment at 5 μM and 10 μM of oleuropein significantly degraded Na_V_1.7 mRNA expression by 1.37-fold and 1.42-fold respectively as well as reduced the cell migration, as determined using Boyden Chamber assay (Aktas and Ayan, 2021).

In addition, anti-invasive effects of two clinically used Na_V_1.7 inhibiting drugs, riluzole and ranolazine were determined in MAT-LyLu cell line at non-toxic doses under normoxic and hypoxic (D’Aiuto et al., 2022) conditions by Rizaner *et al* (Rizaner et al., 2020). The study determined that at 48 h the hypoxia reduced the cell growth by 60%. TTX treatment at 1 μM had no effect on the cell viability under hypoxia and normoxia. Riluzole treatments were safe for MAT-LyLu cells at the concentration up to 5 μM for 48 h in normoxia and 24 h in hypoxia while ranolazine treatment were safe at 20 μM for 24 h in both normoxia and hypoxia conditions. Hypoxia showed no effect on the cell invasion. However, the treatment with TTX significantly decreased the cell invasion in normoxic and hypoxic conditions. Likewise, ranolazine showed a significant reduction in the cell invasion under normoxic and hypoxic conditions. The treatment of riluzole had no effect on the cell invasiveness for up to 24 h in both conditions, however, a decrease in cell invasion was observed at 48 h in normoxic condition. In hypoxia, Na_V_1.7 mRNA expression in MAT-LyLu cells was 400% higher than in normoxia. The riluzole treatment showed no effect on Na_V_1.7 mRNA expression in normoxia but in hypoxia the expression level was suppressed by 43%. Overall, the hypoxic condition had no effect on cell viability and invasion up to 24 h. The treatment of riluzole and ranolazine did not affect MAT-LyLu cell viability up to 24 h. The reduction of MAT-LyLu cell invasion was observed at 24 h after ranolazine treatment and 48 h after riluzole treatment (Rizaner et al., 2020). In summary, Na_V_1.7 isoform is predominantly expressed in prostate cancer and two clinically approved Na_V_1.7 inhibitors, riluzole and ranolazine, have the potential to be used for the treatment of metastatic PCa.

### 5.2 Non-small-cell lung cancer (NSCLC)

A study of mRNA levels of Na_V_1.6 and Na_V_1.7 in human non-small-cell lung cancer (NSCLC) by Roger *et al* published in 2007 determined their expression in cancerous cells, H23, H460, Calu-1, a weakly metastatic cell line, A549 and non-cancerous cell lines, NL-20 and BEAS-2B (Roger et al., 2007). Surprisingly, during the membrane depolarization, inward *I*_Na_ was only observed in H23, H460 and Calu-1 and the protein expression at 250 kDa corresponding to functional channels were also detected in those cell lines. In addition, the internal Na^+^ concentration measured using sodium specific fluorescent probe SBFI, showed a 2-fold increase in the cancer cells, H460 than in the normal NL-20 cells. This effect may be due to the presence of *I*_Na_ between −55 mV and 0 mV in cancer cells which allow Na^+^ influx at its normal membrane potential (−100 to −30 mV) (Roger et al., 2007). The inhibition of *I*_Na_ in H23, H460, and Calu-1 cells upon treatment with 1 μM of TTX did not affect cell proliferation and migration whereas cell invasion was reduced by 40%–50%.

In another study of VGSCs in NSCLC conducted by Campbell *et al* in 2013, the cell line H460 displayed strong cell invasiveness, compared to a more proliferative cell line, A549 which displayed weak cell invasiveness (Campbell et al., 2013). The H460 cells transfected with siRNA against Na_V_1.7 displayed weak cell invasiveness suggesting that Na_V_1.7 is involved in the mechanism of invasion. High expression of Na_V_1.7 mRNA was present in H460 compared to A549 and H460 siRNA. Consistent with the previous results, no *I*_Na_ was detected in A549 compared to H460, which showed a decent *I*_Na_ amplitude with a 2-fold higher intracellular Na^+^ concentration. Na_V_1.7 siRNA completely abolished *I*_Na_ in H460. Furthermore, treatment of H460 cells with TTX shifted the cell resting membrane potential (*E*_m_) to become more hyperpolarized as observed in A549 cells. The TTX treatment also diminished the internal Na^+^ concentration by 50%, while the activation of the channels using veratridine showed no significant effect on an internal Na^+^ concentration. Veratridine enhances sustained inward currents by blocking VGSC inactivation through allosteric mechanism (Cestèle and Catterall, 2000), with a concentration around 29 μM to induce 50% of the maximum effect (Catterall, 1977). The treatment with 0.5 μM TTX or 50 μM veratridine in H460 cells did not affect cell proliferation nor cell migration as determined by wound healing assay. On the other hand, there was a significant reduction in cell invasion where TTX treatment and siRNA transfection of H460 cells showed inhibition of invasion, whereas veratridine treatment increased the cell invasion (Campbell et al., 2013). The expression of Na_V_1.7 in A549 increased the cell invasion by 2-fold suggesting that the functional expression of Na_V_1.7 might be crucial for the invasion activity of NSCLC cells. In addition, the Na_V_1.7 expression in NSCLC patient samples assessed by immunostaining also showed substantially higher expression compared to normal-matched lung tissues (Campbell et al., 2013).

### 5.3 Ovarian cancer (OC)

In 2010, the expression of Na_V_1.1 to Na_V_1.9 were investigated in the highly metastatic ovarian cancer cell lines, Caov-3, SKOV-3, and in a weakly metastatic ovarian cancer cell line, Anglne to correlate their expression to the rate of metastasis (Gao et al., 2010). Based on quantitative PCR experiments, the mRNA for Na_V_1.1, Na_V_1.2, Na_V_1.3, Na_V_1.7 and Na_V_1.8 were detected in all three cancerous cell lines. There were no significant differences in mRNA expression levels of Na_V_1.2, Na_V_1.6, Na_V_1.7 and Na_V_1.9 between cancerous cells and normal ovarian tissues while the expression levels of Na_V_1.1, Na_V_1.3, Na_V_1.4, and Na_V_1.5 were higher in ovarian cancer cells compared to normal ovary cells. When comparing the highly metastatic cells and weakly metastatic cells, significant upregulation of mRNA expression for Na_V_1.2, Na_V_1.4, Na_V_1.5, and Na_V_1.7 were found in highly metastatic cells. The pretreatment with 1 μM TTX did not affect the migration or invasion in Caov-3 and SKOV-3 cells, while the migration and invasion reductions were observed in both cell lines when treated with 30 μM TTX. The reduction of migration and invasion were observed for Caov-3 cells and in SKOV-3 cells. (Gao et al., 2010). This data suggests that the predominant VGSC subtypes in Caov-3 and SKOV-3 cells are more likely to be TTX resistant and the expression levels of Na_V_1.5 and Na_V_1.7 could be correlated with the metastatic activity of ovarian cancer cells.

### 5.4 Gastric cancer (GC)

The effect of Na_V_1.7 on gastric cancer (GC) prognosis was investigated in 2016 by Xia et al. The study found significant correlations between the expression levels of Na_V_1.7, NHE-1 (Na+/H+ exchanger type 1), and MACC1(metastasis-associated with colon cancer-1) in 319 GC patient samples (Xia et al., 2016). Na_V_1.7 and NHE-1 appeared to be highly expressed in patients at advanced metastatic stages, with poor prognosis and lower survival rates. Moreover, Na_V_1.7 was found to be the most abundant VGSC Subtype expressed in GC cell lines, BGC-823 and MKN-28. However, the NHE1 showed variable expression levels where BGC-823 had higher levels compared to MKN-28. Interestingly, cells with higher expression of NHE-1, BGC-823 had larger *I*_Na_ detected with the patch-clamp technique. The inhibition of Na_V_1.7 by using TTX and siRNA targeting *SCN9A* (Na_V_1.7) significantly reduced mRNA and protein expression of NHE-1 and MACC1. The knockdown of *MACC1* gene in both cell lines reduced the expression of NHE-1. In addition, the inhibition of NHE-1 using the NHE-1 inhibitor, 5-(N-ethyl-N-isopropyl) amiloride (EIPA) (Baltazar et al., 2020) and the inhibition of Na_V_1.7 using TTX and siRNA against *SCN9A* caused a reduction in cell proliferation. Regarding cell motility, reductions in cell invasion were observed after treatment with 1 μM TTX, 10 μM EIPA and with the combination of both. In addition, the extracellular pH was found to be significantly higher, while the intracellular pH was lower in the treated groups compared to control. Interestingly, TTX had no effect on invasion under a lower pH condition (pH 6.2) in BGC-823 and MKN-28 cells, suggesting that the intracellular acidic environment could play an important role in cell invasion. Furthermore, an *in vivo* study indicated that the activity of Na_V_1.7 was associated with tumor growth rates. Silencing of Na_V_1.7 (sh-*SCN9A*) in mouse models using BGC-823/sh-*SCN9A* cells showed a depletion of tumor growth. The tumor size and weight were significantly lower in sh-*SCN9A* group for a treatment period of over 25 days. The results of these studies suggest that NHE-1 in GC is a downstream protein in the mechanism and its expression depends on the inhibition cascade of MACC1 and Na_V_1.7.

### 5.5 Endometrial cancer (EC)

In 2019, Liu et al. observed the association of the overexpression of Na_V_1.7 with tumor size, progression and prognosis of endometrial cancer (EC) (Liu et al., 2019). A 25-fold higher mRNA expression for Na_V_1.7 was observed in all 6 EC tissues compared to 6 adjacent non-neoplastic (NE) tissues. To analyze whether Na_V_1.7 expression was correlated with tumor progression and prognosis, the study examined an additional 20 pairs of EC and NE. The results showed that the higher expression of Na_V_1.7 in local lymph node metastasis increased as the tumor size increases. Moreover, patients with high Na_V_1.7 expression had a shorter survival ratio compared to the patients with low Na_V_1.7 expression, suggesting that the Na_V_1.7 expression is associated with the modulation of EC development and invasion. The study further evaluated the anti-cancer effects through the inhibition of Na_V_1.7 using PF-05089771 (Swain et al., 2017) in EC cells and showed an increase in EC cell apoptosis whereas the activation of Na_V_1.7 using veratridine (Zhang et al., 2018) reduced the late apoptosis (Liu et al., 2019). In addition, the treatment with 10 μM TTX for 24 h and 100 μM PF-05089771 significantly decreased EC cells invasion while the treatment of veratridine at the same concentration significantly increased the number of invading cells. The results of this study suggest that Na_V_1.7 activity plays a crucial role in EC progression and metastasis.

### 5.6 Malignant pleural mesothelioma (MPM)

A study of sodium channels in primary malignant pleural mesothelioma (MPM) cells in 2006, by Fulgenzi *et al* revealed the exclusive expression of VGSCs in MPM cells, while it was not expressed in normal mesothelial (NM) cells (Fulgenzi et al., 2006). MPM cells have high expression of Na_V_1.2, Na_V_1.6 and Na_V_1.7 which correlated to the TTX sensitivity with an IC_50_ value for *I*_Na_ inhibition of 16.3 ± 1.9 nM. The treatment of MPM cells with 2 μM of TTX showed no effect on the cell viability and apoptosis, however, this treatment, significantly reduced MPM cell motility (Fulgenzi et al., 2006).

### 5.7 Medullary thyroid cancer (MTC)

More recently, our group reported the expression of Na_V_1.5, Na_V_1.6 and Na_V_1.7 channels in two medullary thyroid cancer (MTC) cell lines: MZ-CRC-1 (metastatic) and TT (primary tumor) using quantitative RT-PCR and immunoblotting assays^48^. We have also demonstrated an elevated Na_V_1.7 channel expression in MTC patients’ tissues compared to normal thyroid tissues (Pukkanasut et al., 2023). The Na_V_1.7 expression was found to be 400-fold higher than Na_V_1.5 and 25-fold higher than Na_V_1.6 in metastatic MTC cell line, MZ-CRC-1; and the trend was similar in primary MTC TT cells. The overexpression of Na_V_1.7 was exclusively detected in MTC over other neuroendocrine tumors (NETs) such as pancreatic NET (BON cells) and pulmonary NET (H727 cells). Moreover, there was no detectable expression of Na_V_1.7 in other types of thyroid malignancies such as papillary (TPC-1 cells) and anaplastic (*H*th7 cells). Among the two MTC lines, the metastatic MZ-CRC-1 showed 2-fold higher mRNA expression of Na_V_1.7 than the primary TT cells. Notably, Na_V_1.7 mRNA expression was significantly upregulated in MTC patient tissues compared to normal counterparts. The Na_V_1.7 expression was observed in four of the six (67%) MTC patient specimens. However, neither Na_V_1.7 mRNA expression nor protein expression was detected in normal thyroid tissues and cell lines, Nthy-ori3-1 and Htori-3. Further, immunostaining of 45 patient samples collected in tissue microarray (TMA) showed consistent results in which Na_V_1.7 expression was more advanced in MTC compared to normal thyroid tissues. In this work, we also identified potent small molecules, SV188 that inhibited sodium channel by evaluating their ability to mitigate mRNA expression of Na_V_1.7 related genes such as *SLC9A1* (NHE-1). The IC_50_ values of SV188 in MTC cell lines were 8.47 μM and 9.32 μM against MZ-CRC-1 and TT, respectively and the IC_50_ of SV188 for *I*_Na_ inhibition of Na_V_1.7 was 3.6 μM in HEK-293 cells transfected with Na_V_1.7. The treatment of 5 μM SV188 inhibited *I*_Na_ in a voltage-dependent manner, with stronger block at *V*_m_ values where *I*_Na_ is outward. SV188 significantly shifted the voltage-dependence of activation of Na_V_1.7 to more negative potentials, however, there was no significant difference on the voltage-dependence of inactivation of Na_V_1.7. The inhibition of Na_V_1.7 by SV188 displayed a use-dependent characteristic. The treatment with SV188 at 3 μM and 6 μM for 48 h inhibited cell migration in both MZ-CRC-1 and TT lines and inhibited cell invasion in MZ-CRC-1 cells. However, SV188 had no effect on weakly metastatic TT cells invasion which has significantly lower expression of Na_V_1.5, Na_V_1.6 and Na_V_1.7 compared to MZ-CRC-1 cells. Cell cycle analysis on MZ-CRC-1 suggested that SV188 inducing a cell cycle arrest at G0/G1 phase. Overall, our data suggests that Na_V_1.7 is uniquely upregulated in MTC and the inhibition of Na_V_1.7 by SV188 showed a use-dependent and a voltage-dependent blockade suggesting that SV188 enters the central cavity of the channel via the intracellular gate and bind within the permeation pathway of the pore channel. SV188 significantly reduced cell motilities and increased cell cycle arrest at G0/G1 phase. This effect suggested that the treatment of MZ-CRC-1 cells with SV188 could have an impact on extenuating cell proliferation.

Overall, the voltage-gated sodium channel α-subunit 1.7 (Na_V_1.7) has been implicated in various cancers due to its role in cancer cell proliferation, migration, and invasion. The experimental data suggests that higher expression of Na_V_1.7 is associated with more aggressive forms of cancers and its activity is linked to cancer cell invasion and migration. The inhibition of Na_V_1.7 either by small molecules at low μM concentrations or Sh-*SCN9A* showed 40%–60% reduction in cell invasion/migration *in vitro* and a reduction in tumor growth rate *in vivo*. Therefore, Na_V_1.7 is a potential drug target for metastatic cancer therapy.

## 6 Proposed mechanism of action of Na_V_1.7 in cancer metastasis

**FIGURE 4:**
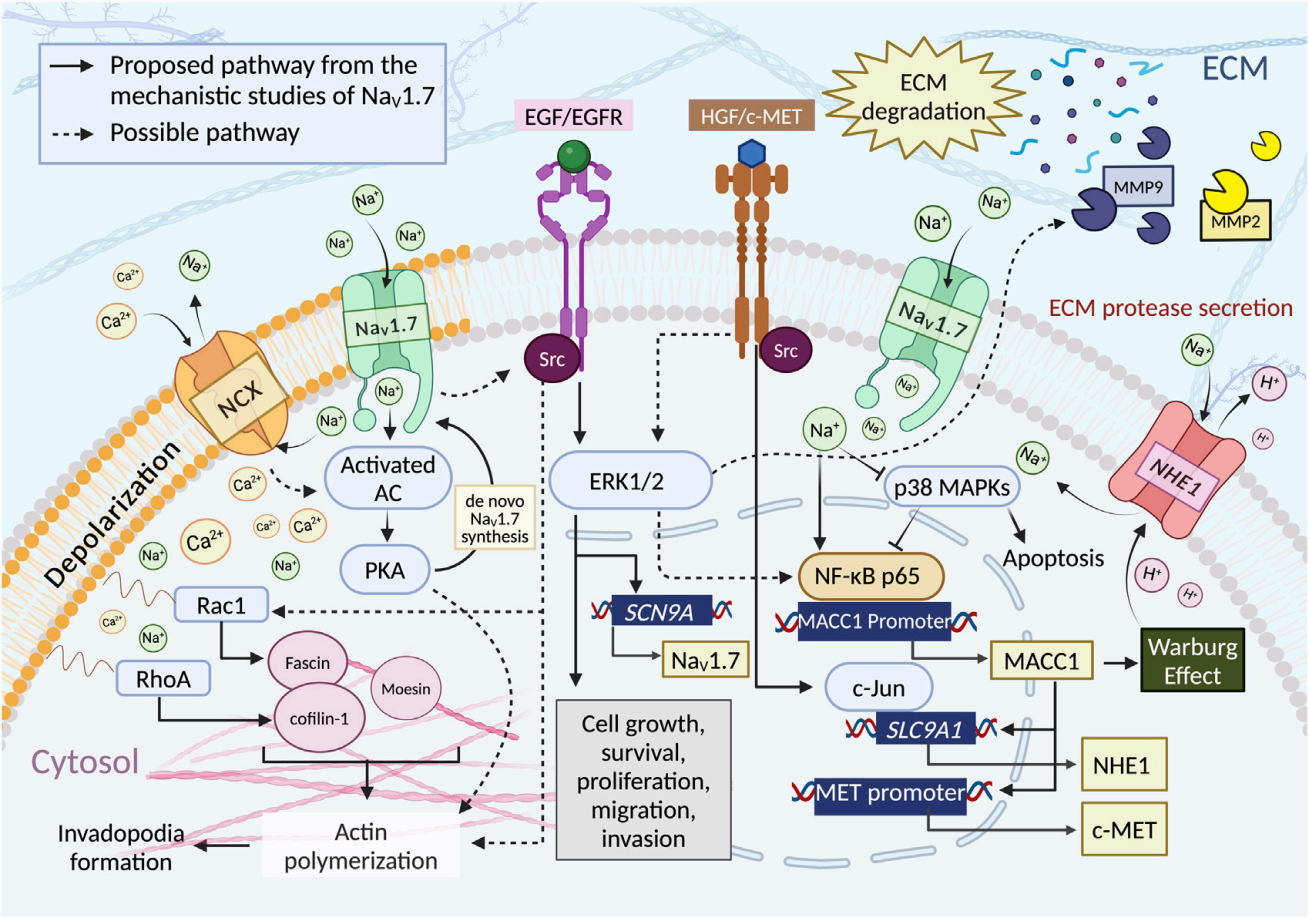
Na_V_1.7 activity is proposed to influence the cancer cell functions such as cell growth, survival, proliferation, and motilities through multiple pathways. i) Intracellular influx of Ca^2+^ ions through the reverse mode of NCX (3:1, Na^+^: Ca^2+^ (Malcolm et al., 2023)). Na_V_1.7 activity increases the intracellular concentration of Na^+^, which promotes Ca^2+^ influx through NCX reverse mode, thus initiating PKA formation through the activated AC. PKA induces actin polymerization by phosphorylation of CIP4, a crucial factor coordinated with an actin polymerization. ii) EGF/EGFR and HGF/c-MET pathway. EGF/EGFR induces the upregulation of Na_V_1.7 expression and enhances the metastasis driving cell functions through ERK1/2 signaling. Evidence showed that ERK1/2 is a downstream target of EGF/EGFR and HGF/c-MET pathway. It could facilitate migration and invasion by regulating the activity of MMPs, iii) Phosphorylation of p38. Na_V_1.7 activity reduces the phosphorylation of p38, which increases the binding of NF-κB p65 to the MACC1 promoter region resulting in an increase of MACC1 expression. The activation of ERK1/2 via HGF/c-MET pathway could also modulate the NF-κB signaling. iv) NHE-1 expression. The expression of NHE-1 depends upon the expression of MACC1, for which c-MET is the transcriptional target. The c-MET binding to HGF activates the interaction of c-Jun with the promoter region of *SLC9A1* to initiate the synthesis of NHE-1. v) Intracellular Na^+^ ion concentration. An Increase in the intracellular concentration of Na^+^ triggers H^+^ efflux through NHE-1 (1:1, Na^+^:H^+^ (Liskova et al., 2019)) leading to an increase in the acidity of ECM environment enhancing MMPs secretion and ECM degradation. NHE-1 activation may also have resulted from the contribution of Warburg effect associated with MACC1. vi) Rho GTPases activity. The regulation of Na_V_1.7 could indirectly increase the Rho GTPases activity. Na_V_1.7 activity causes membrane depolarization and induces Ca^2+^ ion entry through the activation of Cavs and/or reverse-mode NCX, subsequently increase the expression of RhoA and Rac1 to facilitate cell motility by activating Cofilin-1 and Fascin, which are crucial for actin polymerization. Na_V_1.7 activity may enhance Src kinase activity, promoting actin polymerization, cytoskeleton reorganization, and invadopodia formation. Both EGF/EGFR and the activation of HGF/c-MET can activate Src kinases. Src-mediated cancer cell invasion and migration occur through the activation of Rac1. Figure created with BioRender.com.

Even though the mechanism of the involvement VGSCs in cancer progression and metastasis has not been fully resolved, recent literature suggests that the overexpression of different VGSC subtypes facilitate cell motility by increasing ECM protease secretion, cell migration and invasion, invadopodia formation, activation of Src kinases, MAPK kinases, and modulation of Jak/Stat and PKA signaling pathways. These are key processes that enhance cell proliferation, epithelial-mesenchymal transition (EMT) and cell invasion (Brisson et al., 2013; Besson et al., 2015; Roger et al., 2015; Angus and Ruben, 2019; Sanchez-Sandoval et al., 2023). A summary of potential mechanisms of action (MoA) of Na_V_1.7 promoting invasive effects is presented in Figure 4.

### 6.1 PKA activity and Na_V_1.7 expression

In the PCa study using MAT-LyLu cells, the authors have found a reduction of PKA activity as well as the depletion of VGSC protein and *I*_Na_ density in the plasma membrane after TTX pretreatment (Brackenbury and Djamgoz, 2006). Consequently, VGSC protein accumulated in the intracellular compartments, therefore, the total VGSC protein level in the cell was not changed. This evidence suggests that the activity of PKA could potentially be involved in VGSC trafficking and transcriptional processes in order to maintain the amount of VGSC at the plasma membrane (Brackenbury and Djamgoz, 2006).

### 6.2 EGF/EGFR-ERK1/2 enhances motilities via Na_V_1.7

The investigation of the impact of the epidermal growth factor (EGF) on Na_V_1.7 using MAT-LyLu cells under serum free conditions revealed that the cells treated with EGF (100 ng/mL) exhibited a two-fold increase in *I*_Na_ density. However, when cells were co-incubated with the EGFR inhibitor, AG1478 or when an antibody against EGFR was employed, the effect of EGF was abolished and *I*_Na_ density levels decreased significantly compared to the control group (Ding et al., 2008). In terms of cell motility, the use of AG1478 alone or in combination with EGF decreased cell migration. Further studies using 500 nM TTX reduced cell migration whereas the co-treatment with EGF + TTX resulted in a slight increase in cell invasion. These findings suggests that the effect of EGF-meditated cell migration occurs largely through Na_V_1.7 activity (Ding et al., 2008).

Additional study using PC-3M cells showed that the upregulation of functional expression of Na_V_1.7 is related to the concentration of EGF as an increase in EGF concentration resulted in significant upregulation of Na_V_1.7 mRNA expression (Uysal-Onganer and Djamgoz, 2007). Treatment of PC-3M cells with the EGF inhibitor AG1478, reduced Na_V_1.7 mRNA expression and EGF meditated functional basal expression of Na_V_1.7. There was no difference in Na_V_1.7 mRNA expression in EGF + AG1478 co-treated cells vs. AG1478 treatment alone. An increase in cell migration and HRP uptake in a dose dependent manner was observed following the EGF treatments. In addition, EGF treatment significantly increased the cell invasion, while the application of the EGF inhibitor, AG1478 or TTX reduced the cell invasion. The treatment with TTX alone also reduced cell migration and HRP uptake. However, co-treatment TTX with EGF abrogated EGF effect on cell migration and invasion. This data suggests that EGF induces upregulation of Na_V_1.7, enhances the metastatic behaviors such as migration, endocytosis, and invasion and the impact of EGF on cell metastasis was largely facilitated by Na_V_1.7 in PC-3M cells.

The study using the non-small-cell lung cancer cell line, H460 revealed additional information about the relationship between the epidermal growth factor receptor (EGFR) signaling pathway and the expression of Na_V_1.7 mediated through MAP-kinase ERK1/2 (Campbell et al., 2013). EGF/EGFR is associated with four main downstream signaling pathways such as Jak/Stat, PI3-K/Akt, PLC γ and ERK1/2, all of which play important roles in regulating cell growth, proliferation, and ion channel modulation (Oda et al., 2005). Na_V_1.7 expression, mean *I*_Na_ and cell invasion, were decreased by 5-fold upon treatment with an EGFR modulator U0126 which inhibits MAP-kinase ERK1/2 (Zlobina et al., 2015). However, the treatment with wortmannin, an inhibitor of phosphoinositide 3-kinases (PI3Ks) (Sieber et al., 2010) had no effect on Na_V_1.7 mRNA expression, mean *I*_Na_ or invasion. A combined treatment with U0126 and TTX resulted in no significant effect on the cell invasion. These results suggest that the activity of EGF/EGFR-ERK1/2 signaling pathway upregulates Na_V_1.7 functional expression, facilitates *I*_Na_ influx and increases the cell invasion capability in H460 (Campbell et al., 2013).

### 6.3 Na_V_1.7 activity upregulates MACC1 and NHE-1

The study of GC by Xia *et al* in 2016 established a clear relationship between Na_V_1.7, NHE-1 and MACC1 in causing cancer progression. The authors have shown that the suppression of Na_V_1.7 resulted in the reduction of MACC1 and NHE-1 expression (Xia et al., 2016). Further investigation on the mechanism was carried out by assessing the effect on MACC1 expression by inhibiting kinase signaling pathways using a p38 kinase inhibitor SB203580, JNK kinase inhibitor, SP600125, and JAK kinase inhibitor, AG490. Among these, only the p38 kinase inhibitor showed significant increase in MACC1 mRNA expression. Similarly, the inhibition of Na_V_1.7 by TTX and si-*SCN9A* in GC cell lines, BGC-823 and MKN-28 resulted in an increase of p38 mitogen-activated protein kinase (MAPK), a phosphorylated form of p38. These results suggest that the inhibition of Na_V_1.7 increased p38 kinase activity and subsequently reduced the MACC1 expression.

The study further investigated the relationship between the nuclear factor kappa B (NF-κB) activity, p38 MAPK signaling pathways and MACC1 expression. NF-κB was found to be significant in the progression of several human malignancies including genetic and epigenetic alterations, epithelial-to-mesenchymal transition, angiogenesis, invasion, and metastasis (Taniguchi and Karin, 2018). The inhibition of NF-κB-mediated activation of p38 MAPK causes apoptosis in cancer cells while the inhibition of p38 activity induces nuclear translocation and subsequent binding of NF-κB to gene promoters stimulating the tumor progression (Kollipara et al., 2013). The inhibition of Na_V_1.7 using TTX and si-*SCN9A* reduced the binding of NF-κB p65 to MACC1 promoter region and inhibited the translocation of NF-κB p65 into the nucleus by inhibiting the phosphorylation of IκB in both GC cells. Moreover, the treatment with BAY11-7082, a NF-κB inhibitor and the transfection of siRNA NF-κB p65 (si-p65) led to a reduction of MACC1 mRNA and protein levels in both GC cell lines. This data suggests that the inhibition of Na_V_1.7 activated p38 MAPK kinase, inhibited the translocation of NF-κB p65, and subsequently downregulated the expression of MACC1. The results also indicate that inhibition of Na_V_1.7 weakens the NF-κB p65 binding to MACC1 promoter region resulting in the downregulation of MACC1 expression.

The MACC1 meditated regulation of NHE-1 expression was further investigated by focusing on the ability of MACC1 to promote invasion through HGF/c-MET signaling pathway in which c-Jun is a downstream target (Stein et al., 2009). The results showed that the overexpression of MACC1 increased p-c-Jun protein level while the inhibition of c-MET using PF-04217903 led to an overexpression of MACC1 and reduction in phosphorylation of c-Jun (p-c-Jun). In addition, the cells transfected with si-*MACC1* gene showed significantly lower p-c-Jun level whereas the treatment of recombinant human HGF on cells transfected with si-*MACC1* gene observed no significant difference in the p-c-Jun level compared to scramble cells treated with human HGF. Additionally, cells transfected with si-c-Jun significantly reduced NHE-1 protein expression. The activation of Na_V_1.7 in GC cells using veratridine significantly increased p-c-Jun level and NHE-1 expression but, the inhibition of c-MET using PF-04217903 abolished the effect of veratridine. Thereby, c-MET is a transcriptional target of MACC1, and the activity of c-MET can be regulated through HGF. The activity of c-MET triggered c-Jun activation leading to the production of NHE-1. An *in vivo* study showed the silencing of Na_V_1.7 expression (sh-*SCN9A*) reduced the tumor sizes of mice models (Xia et al., 2016). This study showed the upregulation of p38 phosphorylation and the reduction of MACC1, c-MET, p-c-Jun and NHE-1 in sh-*SCN9A* models compared to scramble models using IHC techniques. Taken together, these results suggest that Na_V_1.7 activity in GC increases MACC1 expression through the inhibition of p38 MAPK leading to the regulation of the downstream HGF/c-MET pathway, which meditates c-Jun activity and controls the NHE-1 expression. Moreover, there is a possibility that NHE-1 serves as the downstream target for MACC1 in which NHE-1 activation triggers proton efflux due to Warburg effect and lead to accumulation of lactic acid by a glycolysis metabolism (Birkeland et al., 2020). Lactic acid is associated with the ability of MACC1 to promote the activity and expression of glycolytic enzymes (Lin et al., 2015).

### 6.4 Na_V_1.7 indirectly increases Rho GTPases activity

Results of PCa MAT-LyLu cells treatment with JZTX-I and HNTX-III peptides and 2D electrophoresis screening for the plasma membrane proteins showed 64 differentially expressed proteins associated with Na_V_1.7 regulation and cell motility (Chen et al., 2019). The signaling pathway analysis of these 64 proteins suggested that the modulation of Na_V_1.7 using JZTX-I (activated Na_V_1.7) and HNTX-III (inhibited Na_V_1.7) significantly impacted the carbon metabolism, biosynthesis of amino acids and glycolysis/gluconeogenesis pathways. Eight out of 64 proteins were actin-associated proteins such as annexin A1, annexin A2, confilin-1, fascin, muskelin, moesin, calcyclin-binding protein, and high mobility group protein B1. The migration/invasion associated protein, actin cytoskeleton is known to play an important role in cell adhesion and migration (Staquicini et al., 2017; Devi et al., 2021). The initial step of migration is driven by the actin polymerization, which is catalyzed by Rho GTPases Rac activity (Devi et al., 2021). Rho family GTPase RhoA and Rac1 regulate cell motility through the activation of LIMK1 and the phosphorylation of actin-depolymerizing factor (ADF)/cofilins (Oleinik et al., 2014). All eight proteins were correlated with Rho GTPase-related motility (Adams and Schwartz, 2000; Rescher et al., 2008; Kling et al., 2016). From these eight proteins, fascin and moesin were downregulated while other six proteins were upregulated when inhibited Na_V_1.7 compared to activated Na_V_1.7.

In a separate study, RhoA and Rac1 activities were inhibited using CCG-1423, and EHop-016 (Evelyn et al., 2010; Montalvo-Ortiz et al., 2012). The treatment of MAT-LyLu cells with JZTX-I alone increased the cell invasion while the co-treatment with JZTX-I + CCG-1423 or JZTX-I + EHop-016 diminished the effect of JZTX-I and reduced cell invasion. Similarly, the treatment with HNTX-III alone and co-applied with CCG-1423 or EHop-016, the invasion activity was significantly reduced. Additionally, RhoA and Rac1 basal expression levels increased after treatment with JZTX-I and decreased after treatment with HNTX-III. Moreover, JZTX-I and HNTX-III treatments showed no effect on RhoA and Rac1 expression levels in knocked down Na_V_1.7 MAT-LyLu cells. This data is consistent with the hypothesis that Na_V_1.7 activity is directly related to the invasion capability of PCa MAT-LyLu cells. The regulation of Na_V_1.7 could indirectly affect the downstream Rho GTPases RhoA and Rac1 which then impacted the cell motility.

According to Rao et al. (Rao et al., 2001) and Dulong et al. (Dulong et al., 2014), the activity of RhoA is regulated by intracellular Ca^+^ ion concentration. Increase in Na^+^ influx by Na_V_1.7 increases the Ca^+^ influx through the reverse mode of NCX (Besson et al., 2015) which leads to an increase in intracellular Ca^+^ level that in turn enhances the RhoA expression. It is important to note that inducing membrane depolarization by VGSC has the potential to activate the voltage-gated calcium channels (Cavs) and increase the intracellular concentration of Ca^2+^ ions (Angus and Ruben, 2019). Considering that RhoA and Rac1 are associated with multiple pathways (Clayton and Ridley, 2020; Ma et al., 2023) involved in actin reorganization, cell motility and adhesion such as EGF/EGFR (Wang et al., 2018; Sawaya et al., 2019), Wnt (Jiang et al., 2020; Kim et al., 2022), PAK (Hanna and El-Sibai, 2013; Clayton and Ridley, 2020) and mevalonate (Göbel et al., 2020; Xu et al., 2021) signaling, these pathways could potentially participate in the regulation of the motility of MAT-LyLu cells through the modulation of Na_V_1.7 activity.

### 6.5 Na_V_1.7 activity induces the secretion of MMPs

One of the major factors that modulates metastasis is the tumor ECM environment (Elgundi et al., 2019). From a study of human NSCLC cell lines, it is observed that the blockade of *I*_Na_ by TTX is sensitive only to invasion activity and not to migration (Roger et al., 2007; Campbell et al., 2013). These results suggest that the inhibition of sodium channels suppresses the secretion of ECM digesting proteases such as matrix metalloproteases (MMPs) which might be the proteolytic enzyme controlling the invasive properties of almost every type of human cancers including NSCLC (Egeblad and Werb, 2002). Moreover, the study in GC noticed significant reductions of NHE-1 expression, intracellular pH, and the number of invaded cells when Na_V_1.7 was inhibited by TTX or si-*SCN9A* (Xia et al., 2016). In the study of PCa, PC3 cells showed a distinct reduction in the expression of ECM digesting proteases such as MMP-2 and MMP-9, after the treatment with two small molecule Na_V_1.7 inhibitors, S0154 and S0161(102). This data is consistent with the proposed mechanism in which the activity of Na_V_1.7 triggers proton (H^+^) efflux through NHE-1 and leads to an increase of ECM acidity which enhances the secretion of MMPs by cancer cells (Besson et al., 2015).

### 6.6 Potential mechanistic pathways involving Na_V_1.7

Given that the elevation of intracellular Na^+^ levels impact additional membrane protein exchangers, particularly NCX and NHE-1, it might subsequently influence various other pathways. These pathways intertwine, potentially impacting one another, making them all relevant to cell survival, migration, and invasion activities. As previously noted, higher internal Na^+^ concentration triggers an elevation in intracellular Ca^2+^ concentration and augments the activity of NHE-1. The regulation of Na_V_1.7 could indirectly affect the downstream Rho GTPases RhoA and Rac1 through the depolarization of the cell membrane and the change in intracellular Ca^2+^ concentration (Rao et al., 2001; Dulong et al., 2014).

It is plausible that PKA plays a role in the activity-dependent regulation of Na_V_1.7, however, it has been established that an increase in internal Ca^2+^ concentration also induce PKA production *via* cAMP synthesis, activated by soluble AC (Zhang et al., 2020). Additionally, the study by Dunn *et al* showed that an elevated intracellular Ca^2+^ concentration and membrane depolarization results in PKA activity elevation in retinal ganglion cells (RGCs) (Dunn et al., 2009). Consequently, the levels of PKA activity may rise in response to both intracellular Na^+^ and Ca^2+^ concentrations. Furthermore, PKA has been recognized as a regulator of cell migration. It phosphorylates CDC42 interacting protein 4 (CIP4), a crucial factor which coordinates the actin polymerization and membrane deformation, thereby, promoting cancer cell invasion and metastasis (Zhang et al., 2020).

The Na_V_1.7 expression is involved in the activation of ERK1/2, which has been demonstrated to facilitate GC cell migration and invasion by regulating the activity of downstream proteins such as MMPs (Lei et al., 2022). Further mechanistic investigations have indicated that ERK1/2 serves as the downstream target not only for the EGF/EGFR pathway but also for the HGF/c-MET pathway (Organ and Tsao, 2011; Lei et al., 2022)^,^. It has been demonstrated that the activation of ERK1/2 via HGF/c-MET pathway modulates the nuclear factor kappa B (NF-kB) signaling (Barzaman et al., 2022). Studies have shown that EGFR receptors collaborate with Src, contributing to the induction of EMT progression in cancers (Ortiz et al., 2021). Upon activation, Src kinase interacts with various substrates, initiates downstream signaling which involves p130Cas, oncogenic signaling pathways that promotes Src-mediated cancer cell invasion and migration by modulating cytoskeleton organization through the activation of Rac1 (Liu et al., 2015). Interestingly, evidence suggest that the activation of related VGSC subtype Na_V_1.5 is able to enhance the Src kinase activity to promote actin polymerization, cytoskeleton reorganization, and invadopodium formation (Brisson et al., 2013). It is important to note that, both EGF/EGFR and the activation of c-MET through the binding with HGF can also activate Src kinases (Arnold et al., 2017).

### 6.7 Alteration in intracellular Na^+^ concentration through Na_V_1.7 overexpression

The over expression of Na_V_1.7 would disturb Na^+^ homeostasis in cancer cells as indicated by the elevated intracellular [Na^+^] observed in tumors relative to their normal counterparts (Cameron et al., 1980). The accumulation of Na^+^ tends to regulate diverse physiological adaptations at the cellular levels such as alteration in membrane potential (*V*_m_), pH, and metabolic activity, all of which potentially influence tumor progression (Leslie et al., 2019; Malcolm et al., 2023). The entry of Na^+^ ions induce *V*_m_ depolarization triggering the rearrangement of phospholipid in the plasma membrane. This mechanism promotes the cell proliferation by activating K-Ras dependent MAPK signaling (Zhou et al., 2015) to induce cytoskeletal rearrangements and cell migration (Chifflet et al., 2005) *via* the activation of Rho GTPase Rac1 (Yang et al., 2020). Generally, the extracellular microenvironment of solid tumors exhibits a higher acidity (pH ∼6.8) compared to normal cells (pH ∼7.4). Conversely, the intracellular pH of cancer cells tends to be slightly more alkaline (pH ∼7.6) than normal cells (pH ∼7.2) (Gerweck and Seetharaman, 1996; White et al., 2017). An elevation of Na^+^ gradient across the plasma membrane may impact the pH regulation mechanisms such as the influx of HCO_3_^−^ ions by the cotransporter NBCn1 and the efflux of H^+^ by NHE-1 (Hulikova et al., 2011; Boedtkjer et al., 2013). These processes could contribute to the intracellular alkalization and extracellular acidification of tumor cells. An increase in intracellular Na^+^ concentration may influence Na^+^/K^+^ ATPase activity in order to maintain Na^+^ homeostasis (Sontheimer et al., 1994) which may lead to high energy (ATP) consumption, and an increase in glycolytic metabolism (known as the Warburg effect) (Epstein et al., 2014). Consequently, this metabolic shift could facilitate tumor progression and immunosuppression (Barba et al., 2024). Therefore, the Warburg effect in cancer cells might not be a dysfunction of cellular energetics but rather a physiological adaptation towards the energy demands by the membrane transporter activity. Moreover, there might be a connection between the ion channel activity and the secretion of cytokines and chemokines by cancer-associated fibroblasts (CAFs), which play an important role in cell immunity and metastasis in cancers (Malcolm et al., 2023).

When considering these factors, the activity of Na_V_1.7 has the potential to influence several oncogenic signaling pathways that regulate cancer cell growth, invasion, invadopodia formation, and metastasis. This impact could occurs through i) an increase of intracellular Ca^2+^ concentration causing the activation of PKA and inducing the expression of RhoA and Rac1, ii) EGF/EGFR activation leading to the activation of ERK1/2 pathway and Src kinase activation, iii) upregulating the expression of MACC1, which enhances the Warburg effect, c-MET synthesis, and NHE-1 formation, consequently activating ERK1/2, Src kinase, and increasing the ECM protease secretion and iv) an increase in intracellular [Na^+^] can depolarize *V*_m_, disrupt pH balance and alter metabolic activity in cancer cells.

## 7 Therapeutic potential of targeting Na_V_1.7

Na_V_1.7 is expressed in multiple cancer types, and its expression appears to be correlated with oncogenic progression from normal to primary and metastatic stages. Differences in membrane potential (*V*_m_) and intracellular sodium concentration between cancer cells and normal cells (Yang and Brackenbury, 2013), along with the presence of window sodium current in cancer cells that allows Na^+^ influx during resting potential, suggest that Na_V_1.7 activity in cancer cells is higher compared to normal cells. The activation of Na_V_1.7 using veratridine and JZTX-I promoted cell migration and invasion. Conversely, the inhibition of Na_V_1.7 using small molecule inhibitors has been shown to reduce cancer cells functions in various pre-clinical models, including decrease in cells migration, invasion, and proliferation *in vitro* as well as reductions in tumor formation and metastasis *in vivo*. This suggests that Na_V_1.7 could be a novel molecular target for cancer treatment. Non-specific VGSC inhibitors, which are state-dependent and use-dependent, could be more beneficial for treating cancer types with high expression of multiple VGSC subtypes such as PCa, OC, MPM and MTC. On the other hand, for cancer types that exhibit uniquely higher expression of Na_V_1.7 compared to other subtypes, such as GC and EC, utilizing selective Na_V_1.7 inhibitors targeting VSDII and VSDIV binding sites (Payandeh and Hackos, 2018; Mulcahy et al., 2019) would be more appropriate. An advantage of such approach is to circumvent toxicity arising from the off-target binding to Na_V_ isoforms, especially cardiovascular side effects resulting from the inhibition of Na_V_1.5 and Na_V_1.8, which are expressed in heart muscle (Bagal et al., 2014; Fux et al., 2018; Kitano and Shinozuka, 2022). Therefore, to provide the right treatments for the right patient, tumor marker testing is necessary for better outcomes in targeted therapy (Moore and Guinigundo, 2023). Before administering such drugs, screening patients’ specimens for Na_V_1.7 and other subtypes using IHC, or qRT-PCR might be useful. Na_V_1.7 is primarily known for its role in pain signal transmission. Targeting Na_V_1.7 with selective inhibitor for cancer treatment may offer greater benefit, as this strategy not only inhibits cancer metastasis but also relieves cancer pain associated with treatments such as chemotherapy-induced neuropathic pain (CINP) (Quintão et al., 2019) and/or pain from the disease itself (Eijkelkamp et al., 2012). The cohort study on oxaliplatin-induced peripheral neuropathy (OXAIPN) in patients with gastric, pancreatic, and biliary cancers revealed that individuals with *SCN9A* rs6746030 (Na_V_1.7) and *SCN10A* rs12632942 (Na_V_1.8) polymorphic variants developed chronic OXAIPN or experienced greater severity of chronic OXAIPN (Palugulla et al., 2017). Furthermore, a recent clinical study indicated that the use of a specific type of VGSC inhibitor, antiarrhythmic, showed significant improvement in cancer specific survival (Fairhurst et al., 2023). Therefore, additional research on the use of VGSC inhibitors in cancer patients is required to establish a true connection between VGSC inhibition and cancer progression during the clinical phase.

## 8 Conclusions and future perspectives

VGSC subtype Na_V_1.7 is known to play a crucial role in the initiation and propagation of action potentials during the transmission of pain signals. Although Na_V_1.7’s primary role is in the nervous system, there is emerging evidence suggesting its involvement in progression and metastasis of different cancers. This intricate involvement of Na_V_1.7 in cancers underscores its significance as a potential therapeutic target. Several studies have noted that the upregulation of Na_V_1.7 in aggressive cancer types correlates with metastatic progression and poor prognosis. Multiple mechanisms have been proposed for the activity of Na_V_1.7 in cancer cells. The function of Na_V_1.7 is associated with several signaling pathways that play crucial roles in cell proliferation, migration, invasion, and actin polymerization. This includes pathways such as PKA signaling, EGF/EGFR-ERK1/2 signaling, and Rho GTPases Rac activity. Additionally, Na_V_1.7 activity influences the expression of MACC1 and NHE-1 which are regulated by p38 MAPK kinase, HGF/c-MET signaling, and c-Jun activity. Moreover, Na_V_1.7 activity is associated with the secretion of ECM proteases MMPs in cancer cells. The inhibition of Na_V_1.7 using small molecule drugs or by using si/shRNA has provided promising anticancer activities such as decrease in cancer cell migration, invasion, endocytosis activity and the reduction in tumor growth rate. Thus, targeting Na_V_1.7 to regulate its function and expression using small molecule antagonists and/or genetic engineering is a promising approach to develop cancer therapy that aims to slowdown tumor growth and/or reduce metastatic progression. The use of Na_V_1.7 inhibitors to complement existing cancer therapies may enhance their efficacy. Nevertheless, a more comprehensive investigation into the transcriptional genes affected by Na_V_1.7, which may meditate proliferation and invasion through signaling pathways such as MAPK, Wnt and JAK-STAT is essential to elucidate the underlying molecular mechanisms, develop targeted therapies, and assess the feasibility and efficacy of such clinical interventions.

## Figures and Tables

**Table:** 

**ADC**	(Antibody-drug conjugate)
**BIG**	(Beneath the intracellular gate)
**BVP**	(Bupivacaine)
**Cavs**	(Voltage-gated calcium channels)
**CBZ**	(Carbamazepine)
**CIP**	(Congenital indifference to pain)
**c-MET**	(Mesenchymal-epithelial transition factor)
**Cryo-EM**	(Cryo–electron microscopy)
**CV**	(Central cavity)
**DI**—**D4**	(Domain I—Domain IV)
**DRG**	(Dorsal root ganglion)
**ECM**	(Extracellular matrix)
**EC**	(Endometrial cancer)
**EGF**	(Epidermal growth factor)
**EGFR**	(Epidermal growth factor receptor)
**EIPA**	(5-(N-ethyl-N-isopropyl) amiloride)
***E***_**m**_	(Resting membrane potential)
**EMT**	(Epithelial-mesenchymal transition)
**EPA**	(Eicosapentaenoic acid)
**ERK1/2**	(Extracellular signal-regulated kinase ½)
**F**	(Fenestration site)
**G**	(Intracellular activation gate)
**GC**	(Gastric cancer)
**h**	(Hours)
**H-bonding**	(Hydrogen bonding)
**HDA**	(Hardwickiic acid)
**HGF**	(Hepatocyte growth factor)
**HRP**	(Horseradish peroxidase)
**I**_***Na***_	(Sodium current)
**JZTX-I**	(Jingzhaotoxin-I)
**LA**	(Local anesthetic)
**LCM**	(Lacosamide)
**LTG**	(Lamotrigine)
**MACC1**	(Metastasis-associated with colon cancer protein 1)
**MAPK**	(Mitogen-activated protein kinase)
**mCRPC**	(Metastatic castration-resistant prostate cancer)
**MPM**	(Malignant pleural mesothelioma)
**MTC**	(Medullary thyroid cancer)
**mV**	(Millivolt)
**MW**	(Molecular weight)
**NCX**	(Sodium calcium exchanger)
**NF-κB**	(Nuclear factor kappa B)
**NHE-1**	(Sodium hydrogen exchanger-1)
**NHTX-III**	(Hainantoxin-III)
**NSCLC**	(Non-small-cell lung cancer)
**OC**	(Ovarian cancer)
**PCa**	(Prostate cancer)
**PD**	(Pore forming domain S5-S6)
**PI3Ks**	(Phosphoinositide 3-kinase)
**PLC**	(Phosphoinositide-specific phospholipase C)
**PKA**	(Protein kinase A)
**PNS**	(Peripheral nervous system)
**RLZ**	(Riluzole)
**SF**	(Selectivity filter)
**shRNA**	(Short hairpin RNA)
**siRNA**	(Small interfering RNA)
**TTX**	(Tetrodotoxin)
**VGSC**	(Voltage-gated sodium channel)
**VGCC**	(Voltage-gated calcium channel)
**VGKC**	(Voltage-gated potassium channels)
**VPC**	(Vipocetin)
**VSD**	(Voltage-sensing domain S1-S4)
**VXT**	(Vixotrigine)
**μM**	(Micromolar)
**nM**	(Nanomolar)
